# The influence of antigen targeting to sub-cellular compartments on the anti-allergic potential of a DNA vaccine^☆^

**DOI:** 10.1016/j.vaccine.2013.08.005

**Published:** 2013-12-09

**Authors:** Esther E. Weinberger, Almedina Isakovic, Sandra Scheiblhofer, Christina Ramsauer, Katrin Reiter, Cornelia Hauser-Kronberger, Josef Thalhamer, Richard Weiss

**Affiliations:** a Department of Molecular Biology, University of Salzburg, 5020 Salzburg, Austria; b Deparment of Pathology, University Hospital Salzburg, Paracelsus Medical University, Salzburg, Austria

**Keywords:** Type I allergy, Immunotherapy, DNA vaccine, Ubiquitination, ER-targeting, LIMP-II, AA, amino acid, AHR, airway hyperreactivity, APC, antigen presenting cell, BAL, bronchoalveolar lavage, BALF, bronchoalveolar lavage fluid, LIMPII, 20-amino-acid C-terminal tail of lysosomal integral membrane protein-II, SIT, specific immunotherapy, Th, T helper, tPA, human tissue plasminogen activator leader peptide, Treg, T regulatory, Ubi, ubiquitin

## Abstract

**Background:**

Gene vaccines offer attractive rationales for prophylactic as well as therapeutic treatments of type I allergies. DNA and mRNA vaccines have been shown to prevent from allergic sensitization and to counterbalance established allergic immune reactions. Recent advances in gene vaccine manipulation offer additional opportunities for modulation of T helper cell profiles by specific targeting of cellular compartments.

**Methods:**

DNA vaccines encoding the major birch pollen allergen Bet v 1.0101 were equipped with different leader sequences to shuttle the antigen to lysosomes (LIMP-II), to trigger cellular secretion (hTPA), or to induce proteasomal degradation via forced ubiquitination (ubi). Mice were pre-vaccinated with these constructs and the protective efficacy was tested by subcutaneous Th2-promoting challenges, followed by allergen inhalation. IgG antibody subclass distribution and allergen-specific IgE as well as cytokine profiles from re-stimulated splenocytes and from BALFs were assessed. The cellular composition of BALFs, and lung resistance and compliance were determined.

**Results:**

Immunization with all targeting variants protected from allergic sensitization, i.e. IgE induction, airway hyperresponsiveness, lung inflammation, and systemic and local Th2 cytokine expression. Surprisingly, protection did not clearly correlate with the induction of a systemic Th1 cytokine profile, but rather with proliferating CD4+ CD25+ FoxP3+ T regulatory cells in splenocyte cultures. Targeting the allergen to proteasomal or lysosomal degradation severely down-regulated antibody induction after vaccination, while T cell responses remained unaffected. Although secretion of antigen promoted the highest numbers of Th1 cells, this vaccine type was the least efficient in suppressing the establishment of an allergic immune response.

**Conclusion:**

This comparative analysis highlights the modulatory effect of antigen targeting on the resulting immune response, with a special emphasis on prophylactic anti-allergy DNA vaccination. Targeting the antigen to proteasomal or lysosomal degradation reduces the availability of native allergen, thereby rendering the vaccine hypoallergenic without compromising efficacy, an important feature for a therapeutic setting.

## Highlights

• DNA vaccine targeting affected humoral and cellular immunity.
• Elevated Th1 immunity did not correlate with superior protection from sensitization.
• Plasmid vaccination boosted Treg numbers within re-stimulated splenocyte cultures.

## 1 Introduction

One of the unique features of gene vaccines is their potential to design optimized immunization approaches specifically tailored for a wide range of diseases including cancer [1], infectious diseases [1,2], autoimmune diseases [3] and allergic disorders [4,5]. Since their first description in the early 1990s, substantial efforts have been made to enhance the immunogenicity of gene vaccines and to instruct the proper branch of the immune defense, depending on the type of pathogen/disease. Among the numerous ways how to modulate translation of antigens, are strategies to shuttle the antigen of interest to different subcellular compartments (cytoplasm, endosomes/lysosomes [6–9]), to induce cellular secretion [10,11] or cell membrane insertion [12], or guiding to protein-processing machineries (proteasome [13–17], endosome/lysosome). All these modifications are part of mechanisms that play an important role in host-pathogen interactions and represent evolved strategies for optimizing immune responses.

Our working group has specialized on genetic vaccination against allergy and we, as well as others, could provide evidence that plasmid-encoded antigens trigger the induction of a Th1-balanced immune profile [18–20] that is capable of counterbalancing and protecting from allergic sensitization [21,22]. All the mentioned approaches influence the immunogenicity and balance of humoral/cellular immunity; moreover, some of them act on T-helper cell polarization (Th1, Th2, Th17, Treg). The latter aspect plays a crucial role in the development of both, protective as well as therapeutic gene vaccination approaches against type I allergy.

Here, we compared a compendium of DNA vaccine targeting strategies (Fig. 1) on major birch pollen allergen Bet v 1.0101 (Bet) specific allergy. DNA vaccines were constructed with the allergen gene linked with sequences encoding the (i) human tissue plasminogen activator leader peptide (tPA), (ii) ubiquitin (Ubi), (iii) the 20AA C-terminal tail of the lysosomal integral membrane protein-II (LIMPII), or (iv) left without modification. The tPA leader sequence encodes a strong signal peptide for protein secretion thus mediating the release of antigen into the extracellular space. Secreted antigen can be taken up and processed by APC (Fig. 1[1a]), resulting in presentation of antigenic peptides on MHC-II. Nevertheless, a small part will be presented on MHC-I molecules by a mechanism which is called cross-priming [14]. In contrast, Bet lacking a secretory signal sequence, is expressed as cytoplasmic protein (Fig. 1[1b]) and will be presented on MHC-I per default. Like with crosspriming, which ensures antigen delivery from the MHC-II pathway to MHC-I, mechanisms have evolved which enable the exchange of molecules from MHC-I to MHC-II pathways. These mechanisms include shedding of native protein from transfected cells by a still unknown mechanism, which was described as “leakage” (Fig. 1[2]), release of antigen within apoptotic vesicles (immune apoptosis), and transport of cytosolic material into the MHC-II pathway via autophagy (Fig. 1[3]) [23,24].

Ubiquitination shuttles the antigen into the polyubiquitination pathway (Fig. 1[1c]), thereby specifically promoting the presentation of antigenic peptides on MHC-I [17]. In contrast, LIMPII peptide attachment facilitates the antigenic transport from the cytosol to lysosomes (Fig. 1[1d]), thus allowing MHC-II presentation [6].

Our data indicate that targeting has a substantial effect on the strength of humoral immunity, and all targeting variants demonstrated a Th1-bias. Furthermore, targeting proved to be a valuable approach to develop new rationales for optimized anti-allergic gene vaccines.

## 2 Materials and methods

### 2.1.1 Mice, treatment schedules and serology

Female, 6–10 week-old BALB/c mice were obtained from Charles River Laboratories (Sulzfeld, Germany). All animal experiments were conducted according to local guidelines approved by the Austrian Ministry of Science and in accordance with EU Directive 2010/63/EU.

To assess humoral/cellular profiles after vaccination, BALB/c mice (*n* = 5) were immunized intradermally (i.d.) with plasmid DNA encoding Bet, Ubi-Bet, tPA-Bet or Bet-LIMPII on days 0, 7 and 14. 100 μg plasmid DNA in 200 μl sterile PBS were i.d. injected at 6–8 spots on the back of isoflurane-anesthetized animals. On day 49, blood samples were taken and splenocytes were prepared (Fig. 2A). To analyze the protective efficacy of the vaccine on alum-induced allergic sensitization (Fig. 3A), vaccinations on days 0, 6, and 13 were performed as described above. On days 27, 41 and 48, mice were sensitized by two subcutaneous (s.c.) injections with 5 μg of recBet v 1.0101 (Biomay AG, Vienna, Austria) in 100 μL PBS emulsified in 100 μL Al(OH)_3_ (Alu–Gel–S, Serva). On days 57–59, mice received airway challenges by exposure to aerosolized Bet protein in PBS (1 mg/mL) using a jet nebulizer (PARIBOY^®^LCplus; PARI GmbH, Starnberg, Germany). 24 h later, animals were sacrificed after invasive lung measurement of lung functions, and bronchoalveolar lavages (BALs) were collected. Antigen-specific serum IgG1, IgG2a, and IgE were determined by ELISA or RBL assay, and cell-bound IgE was detected by a basophil activation test (BAT). A detailed description of these and additional methods can be found in supplementary materials online.

## 3 Statistical analysis

Statistical significance between groups was assessed by Students *t*-test and correlations were assessed by Spearman's Rank Correlation using GraphPad Prism 5.01.

## 4 Results

### 4.1 In vitro expression of targeting variants

To assess the targeting efficacy of vaccine-derived antigen to different subcellular compartments, BHK-21 cells were transfected in vitro with plasmid DNA encoding Bet, eGFP or the targeting versions of these molecules (tPA-Bet, tPA-GFP, Ubi-Bet, Ubi-GFP, Bet-LIMPII or GFP-LIMPII). Secretion (tPA-GFP) as well as forced proteasomal degradation (Ubi-GFP) of the gene products significantly reduced the cellular mean fluorescence intensity (MFI) of eGFP compared to the unmodified version (Suppl. Fig. 2A). Attachment of the LIMPII peptide increased the MFI, surprisingly, as endosomal targeting should reduce the fluorescence of eGFP by both – proteolytical degradation as well as lowered pH.

Western blot analysis of Bet targeting construct transfections confirmed cytosolic Bet protein expression at 17.6 kDa, Bet-LIMPII at 19.8 kDa and tPA-Bet at 20.7 kDa (Suppl. Fig. 2B). tPA-Bet contained two putative signal peptide cleavage sites as predicted by the SignalP 4.0 algorithm [25], located between AA22-23 and AA28-29, resulting in a molecular weight (MW) of 18.46 kDa or 17.9 kDa for Bet protein without tPA leader, respectively. Analysis of the supernatant of transfected cells confirmed successful Bet protein secretion (Suppl. Fig. 2B).

Ubiquitinylated Bet protein (Ubi-Bet) displayed as two bands (26.4 kDa and 17.6 kDa), representing Bet protein with and without ubiquitin, although we introduced a Gly76–Ala76 point mutation to diminish the cleavage rate of the fusion protein [26]. Successful polyubiquitination was observed as bands of increasing MW (Suppl. Fig. 2B).

### 4.2 Selective targeting of DNA vaccine-derived Bet v 1.0101 to specialized subcellular compartments modulates the Th1-polarized immune response

Modulation of the Bet-specific humoral and cellular immunity was addressed using the experimental design shown in Fig. 2A. Vaccination of BALB/c mice with plasmid DNA encoding Bet was characterized by substantial humoral and cellular memory immune responses [27]. High titers of Bet-specific IgG1 and the induction of IgG2a, as well as IFN-γ producing splenocytes upon in vitro Bet protein re-stimulation, both of which are indicative for Th1 immunity, could be detected 5 weeks after i.d. application (Fig. 2B, C and E). Ubiquitination of Bet (Ubi-Bet) abrogated antibody responses due to immediate epitope destruction, but still induced a potent Th1-polarized immune response (Fig. 2B–E). A similar effect was observed for lysosomal targeting using Bet-LIMPII. Secretion of Bet (tPA-Bet) led to comparable levels of IgG1/IgG2a as observed for Bet-vaccinated animals (Fig. 2B and C), but the cellular response was significantly enhanced (Fig. 2D and E). This is consistent with published data showing that tPA-mediated secretion enhances immunogenicity against plasmid DNA-derived proteins [11]. Therefore, we next tested the capacity of Bet targeting variants to protect from alum-induced Bet-specific sensitization (Fig. 3A).

### 4.3 Bet targeting variants protect from induction of IgE by allergic sensitization with recombinant Bet v 1.0101

Sham immunized (mock) and non-immunized control animals (control) displayed the typical picture of sensitization with alum adsorbed allergen, including high IgG1 and IgE titers as well as the absence of IgG2a (Fig. 3B–E). Pre-vaccination with targeting variants resulted in the reduction of Bet-specific IgG1 (Fig. 3B), boosting of IgG2a (Fig. 3C) and the potent blockage of Bet-specific IgE induction (Fig. 3D). Flow cytometric analysis of blood basophils via basophil activation test (BAT) confirmed a reduced Bet protein-induced activation of basophils in tPA-Bet and Bet-LIMPII pre-vaccinated animals (Fig. 3E). Noteworthy, while the RBL assay is a direct read out of allergen-specific IgE and non-cell bound IgGs are washed away before addition of antigen, the stimulation of basophils during the basophil activation test takes place in whole blood samples. Therefore, blocking IgGs can compete for antigen binding sites and interfere with IgE cross-linking on basophils. To determine, whether immunization with our Bet construct also induced blocking antibodies, we performed a BAT in the presence or absence of antibody containing plasma. Indeed, removal of plasma from PBMCs increased activation of basophils from Bet immunized mice. These data indicate the presence of blocking antibodies in plasma of Bet vaccinated mice, but not in sensitization controls (Suppl. Fig. 3).

### 4.4 Pre-vaccination with Bet targeting variants specifically suppresses Th2-associated cytokine production and increases the percentage of FoxP3+ CD25+ cells in Bet re-stimulated proliferating CD4+ T cells

Analysis of Bet re-stimulated splenocytes revealed a downregulation of Th2 immune responses in all pre-vaccinated groups, indicated by drastically reduced IL-4 spot forming units (SFU) (Fig. 4A) as well as IL-5 (Fig. 4B) and IL-13 (Fig. 4D) in the supernatants of splenocytes. tPA-Bet was less effective in IL-13 suppression than the other targeting variants, although increased numbers of IFN-γ producing splenocytes could be detected after vaccination (Fig. 2E) and sensitization (Fig. 4A). Noteworthy, the number of IFN-γ-producing cells primed by pre-vaccination for Bet and the other targeting variants (Ubi-Bet and Bet-LIMPII) were minimally boosted by sensitization and did not differ from mock or control (Fig. 4A).

Pre-vaccination also suppressed IL-22 (Fig. 4C), indicative for Th17/Th22 cells [28] that may contribute to the early onset of allergic lung inflammation. Again, tPA-Bet was less effective in inhibiting IL-22 production. Pre-vaccination also inhibited IL-6 (Fig. 4F), another cytokine contributing to enhanced Th17 differentiation [29].

Flow cytometric analysis of CFSE-stained splenocytes showed that pre-vaccination diminished the proliferative potential of Bet re-stimulated CD4+ T cells (Fig. 4H) a feature associated with successful plasmid DNA vaccination [14]. Further analysis of proliferating CD4+ T cells in splenocyte cultures revealed a strongly elevated percentage of CD25+ FoxP3+ cells within this pool (Fig. 4I), compared to control and mock, but decreased levels of IL-10 (Fig. 4E).

### 4.5 Bet targeting variants suppress lung inflammation and airway hyperreactivity

Lung inflammation and airway hyperreactivity (AHR) induced upon consecutive Bet protein inhalation were analyzed 24 h after the last allergen challenge by flow cytometric analysis and invasive lung measurement. Pre-vaccination with Bet abrogated AHR reactions, as measured by airway resistance (Fig. 5A) and compliance (Fig. 5B). Ubi-Bet and Bet-LIMPII were slightly less effective in reducing AHR parameters, while tPA-Bet completely failed to provide protection. Nevertheless, collected BALFs showed significantly reduced numbers of infiltrating leukocytes (Fig. 5C), especially eosinophils (Fig. 5D), in all pre-vaccinated groups. The MHC-I and MHC-II targeting variants turned out to be most effective in inhibiting CD4+ and CD8+ lymphocytic infiltration, while unmodified Bet and tPA-Bet could only reduce the influx of CD4+ T cells (Fig. 5E). Bet-LIMPII was also the most effective variant in suppressing neutrophils (Fig. 5F) and macrophages (Fig. 5G). Corresponding to the total numbers of CD4+ T cells, also the number of CD4+ FoxP3+ cells was diminished, however in contrast to the data from re-stimulated splenocytes (Fig. 4I) the percentage of FoxP3+ T helper cells was similar in all groups (Bet 14.5 ± 1.3%, Ubi-Bet 15.6 ± 1.4%, tPA-Bet 15.1 ± 0.96%, Bet-LIMPII 13.1 ± 1.1%, control 15.5 ± 3.2%, mock 13.8 ± 0.7%).

In line with eosinophil and lymphocyte data, Th2-associated cytokines such as IL-4, IL-5, and IL-13 were also reduced in all vaccinated groups (Fig. 5H–J). Th1 (IFN-γ, IL-2, TNF-α), Treg (IL-10) and Th17/Th22 (IL-17, IL-22) associated cytokines as well as, IL-1α, IL-21, and IL-27 did not differ among all groups or were beyond detection limit (IL-17).

To confirm that the suppression of cellular infiltrates in BALFs reflected the lung tissue in situ, we exemplarily compared the cellular composition of collagenase digests of the right lung lobes of Bet immunized mice compared to sensitization controls and naïve mice in a separate experiment. As shown in supplementary Fig. S4, vaccination suppressed the numbers of tissue eosinophils (Suppl. Fig. 4B) as well as alveolar macrophages (Suppl. Fig. 4D), and there was a high correlation between eosinophil numbers from BALF and tissue digest (Suppl. Fig. 4E, *P* < 0.0001). We also addressed the question, whether the suppression of airway inflammation was associated with a change in GITR-L expression on antigen presenting cells in the lung. When staining with CD11b and CD11c we observed CD11b^high^ CD11c^low^ cells (Suppl. Fig. 5A, upper left quadrant) that corresponded to neutrophils, a population of CD11b^high^ CD11c^high^ cells (Suppl. Fig. 5A, upper right quadrant) that most likely represented freshly recruited alveolar macrophages [30], and CD11b^med^ CD11c^high^ cells (Suppl. Fig. 5A, lower right quadrant) that were probably pulmonary DCs. Sensitization induced a strong influx and/or maturation of CD11b^high^ CD11c^high^ alveolar macrophages (Suppl. Fig. 5B) as described in pneumococcal infection [30], while the number of pulmonary DCs was only slightly enhanced (Suppl. Fig. 5C). While GITR-L expression on pulmonary DCs was similar to naïve mice (Suppl. Fig. 5E), alveolar macrophages in sensitized mice, but not in Bet pre-vaccinated mice, showed an upregulation of surface GITR-L (Suppl. Fig. 5D).

The left lung lobes of the same mice were also analyzed histologically on HE and PAS stained paraffin sections. Overall, there was only weak to moderate tissue inflammation (Suppl. Fig. 6); however, pre-vaccination with Bet reduced lung pathology compared to sensitization controls. PAS staining revealed no mucus-producing goblet cells in any of the samples (not shown).

## 5 Discussion

Genetic immunization harbors great potential for the development of human vaccines, as demonstrated by numerous preclinical and clinical studies treating a variety of diseases ranging from viral infections to cancer. The potential to induce Th1-balanced immunity against encoded antigens renders gene vaccines interesting candidates, particularly for prophylactic allergy vaccination. Such an approach demands careful selection of allergens to be included and close consideration of the timing of intervention [31]. Recently, several biomarkers have been established in order to predict the risk for sensitizations to allergens [32–34], which may allow for prophylactic allergy treatment in genetically predisposed, presymptomatic individuals. Clearly, prophylactic treatment requires highest safety standards and thorough knowledge of immunomodulatory consequences. In the current study we provide a comprehensive comparison of DNA vaccine vector modifications feeding the encoded allergen into selected subcellular compartments (Suppl. Fig. 1), thereby modulating the resulting immune response. In line with previous publications we could demonstrate effective targeting of cellular compartments by appending the respective leader sequences (Suppl. Fig. 2), except of LIMPII. In contrast to Rodriguez et al. [7], in our hands, BHK-21 transfections using GFP-LIMPII did not result in co-localization of the GFP signal with lysosomal markers (data not shown) or lysosomal degradation (Suppl. Fig. 2). However, Bet-LIMPII displayed a severely reduced capacity to induce antibody-responses (Fig. 2B and C), indicating a more potent intracellular degradation compared to the cytosolic (Bet) or the secreted Bet (tPA-Bet). Therefore, the effect of the LIMPII targeting sequence may be antigen-dependent.

In vivo evaluation of the anti-allergic capacity of Bet targeting variants in a mouse model of allergy revealed potent regulatory potential for the induction of cellular and humoral immunity. Particularly, targeting of proteolytical compartments (lysosome) or machineries (proteasome) proved to be a limiting factor for the induction of humoral immunity (Fig. 2B and C). This may provide a general approach to reduce the amount of protein in its native conformation, i.e. the availability of B cell epitopes, thereby reducing the risk of unwanted antigen-antibody complex-mediated side reactions upon vaccination. Simultaneously, immunogenicity on T cell level was retained (Fig. 2E and F). All constructs maintained their ability to block IgE induction upon sensitization (Fig. 3D), a necessary prerequisite for anti-allergy DNA vaccination.

We and others have previously demonstrated that the anti-allergic capacity of plasmid DNA and mRNA vaccines strongly correlates with promotion of Th1-biased responses, and is dependent on IFN-γ and in part on IL-12 [35–37]. On the other hand, induction of T regulatory cells via nucleic acid vaccination has also been shown [38]. Although in our current work, all targeted vaccines induced Th1-biased immune responses (Fig. 2C and E), the Th1 memory responses after sensitization (Fig. 4A) did not correlate with suppression of Th2 immunity (Fig. 4A, B and D) and lung pathology (Fig. 5). Although guiding the antigen to the secretory pathway (hTPA) led to enhanced Th1 immunity, this group showed the weakest suppression of systemic and local Th2 cytokines and no protection from allergen-induced AHR. Notably, also sham-immunized and non-immunized control mice displayed elevated numbers of IFN-γ secreting T cells after sensitization (Fig. 4A). Recent publications highlighted the induction of IFN-γ producing CD8+ T cells by alum-absorbed protein [39,40] along with Th2-polarized CD4+ T cells, substantiating our observation of IFN-γ production in ELISPOT for these experimental groups. Interestingly, tPA-Bet vaccinated mice also displayed lower numbers of splenic CD25+ FoxP3+ regulatory T cells compared to the other DNA vaccinated groups (Fig. 4I), indicating the importance of this cell type in our model. The percentage of CD25+ FoxP3+ T cells inversely correlated with the amount of secreted IL-2 in culture supernatants (*P* < 0.001), which may indicate competition for IL-2 secreted by responder T cells, a mechanism by which Tregs can also exert their suppressive potential [41]. Although IL-10 is an important effector cytokine secreted by Treg cells for active suppression [42], in our model IL-10 secretion was reduced in pre-vaccinated animals (Fig. 4E). Indeed, the secretion of IL-10 by re-stimulated splenocytes clearly correlated with the secretion of IL-4, IL-5, and IL-13 (*P* < 0.0001) suggesting that it was Th2 cell-derived. Pre-vaccination also suppressed IL-22 secretion, an indicator of Th17 polarization. Although we could not detect IL-17, sensitization can lead to the generation of IL-22 secreting Th17 cells via the inflammasome/IL-1β axis [43].

Allergen-specific blocking IgG antibodies, which occupy the binding sites for IgE on allergens and control basophil activation via low-affinity IgG receptors [44] represent another mechanism of successful immune therapy [45,46]. Production of allergen-specific blocking IgG has been previously demonstrated following immunization with trimers of hypoallergenic fragments of Bet v 1 [47] or a mimotope gene vaccine [48]. Here, DNA vaccination induced high levels of allergen-specific IgG2a after sensitization, but suppressed IgG1 (Fig. 3B and C). Removal of plasma from PBMCs during the basophil activation test increased the reactivity of basophils in Bet pre-vaccinated mice, but not in sensitization controls (Suppl. Fig. 3), indicating the blocking capacity of the induced antibodies. However, the exact mechanism of how DNA vaccine-induced antibodies exert their blocking effect (via low-affininity IgG receptor mediated suppression or inhibition of IgE binding), remains to be determined.

Aerosol challenge of sensitized mice induced AHR and an influx of leukocytes (mainly eosinophils and T cells) into the lung (Fig. 5). Targeting the antigen to either MHC-I (ubi-Bet) or MHC-II (Bet-LIMPII) proved to be especially effective in suppressing lung recruitment of CD4+ and CD8+ T cells as well as neutrophils, and in the case of Bet-LIMPII, also macrophages. Sensitization induced a clearly Th2-biased cytokine milieu in the lung, which was efficiently suppressed by pre-vaccination with the DNA vaccines, without inducing detrimental effects or changes in the Th1 or Th17 cytokine profile. Interestingly, alveolar macrophages from sensitized, but not from Bet vaccinated mice showed enhanced expression of GITR-L (Suppl. Fig. 5). GITR signaling has been implicated in both, expansion of Treg as well as effector T cells in a context-dependent manner [49]. It has been recently shown, that during Th2-polarized airway inflammation GITR-L was upregulated on lung epithelial cells which in turn lost their potential to suppress local T-cell responses [50]. GITR-L has also been shown to be upregulated on APC during inflammatory processes and to drive inflammation by expansion of effector T cells [51]. Nevertheless, further experiments will be necessary to elucidate the role of alveolar macrophages and GITR-L expression in the initiation and progression of Th2 airway inflammation.

## 6 Conclusions

Although DNA vaccines can prime Th1-biased immune responses and their anti-allergic mechanism has been clearly associated with the induction of IFN-γ, we and others have also demonstrated their potential to induce regulatory T cell responses. Here we show that protection from allergic sensitization and lung inflammation can be achieved without dominant Th1-priming on the systemic (spleen) or local (lung) level, presumably through the antigen-driven expansion of CD25+ FoxP3+ Treg cells. Targeting the antigen to proteasomal or lysosomal degradation was especially potent in reducing inflammatory infiltrates in BAL and simultaneously rendered the vaccine hypoallergenic. In contrast, active secretion from transfected cells proved deleterious on the vaccine‘s protective efficacy. The acquired data will therefore help to construct tailor-made anti-allergy vaccines with increased benefit/risk ratios.

## Funding support

Funding statement: This work was supported by the Austrian Science Fund (FWF), Project # W1213, and the Christian Doppler Research Association. The funders had no role in study design, in collection, analysis, and interpretation of the data, in the writing of the manuscript, and in the decision to submit the paper for publication.

## Author contributions

E.W. and A.I. performed experiments and acquired data. E.W. oversaw the conduct of the study, participated in data interpretation and prepared the manuscript. R.W. and S.S. designed the study, performed experiments, data analysis and interpretation, and edited the manuscript. C.R. gave technical assistance. K.R. performed lung histology and C.H.K. scored lung sections. J.T. supervised the study and edited the manuscript. All authors contributed to revising the manuscript and approved the final version.

## Conflicts of interest

E.W., A.I. and S.S. have received funding from the Austrian Science Fund. R.W. and J.T. have received research support from the Christian Doppler Research Association and from Biomay AG, Vienna, Austria.

## Figures and Tables

**Figure:**
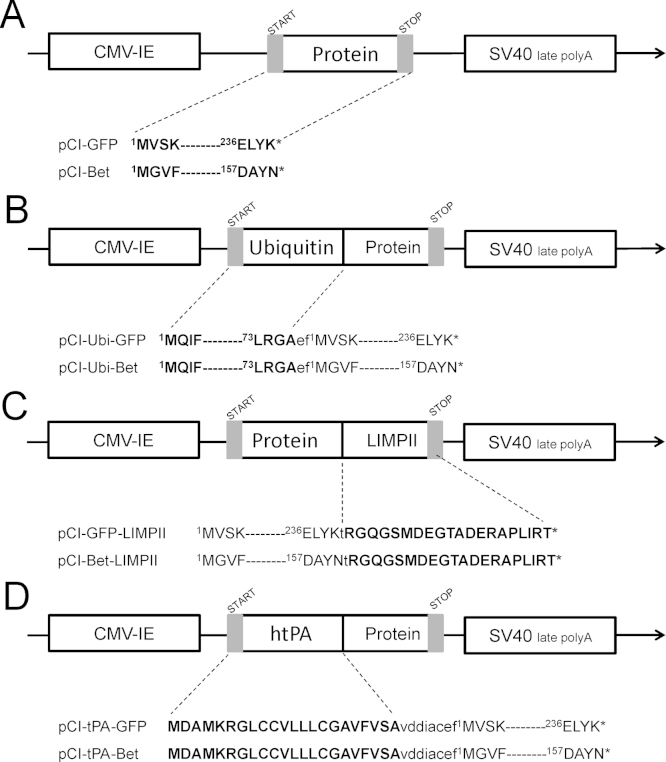


**Figure:**
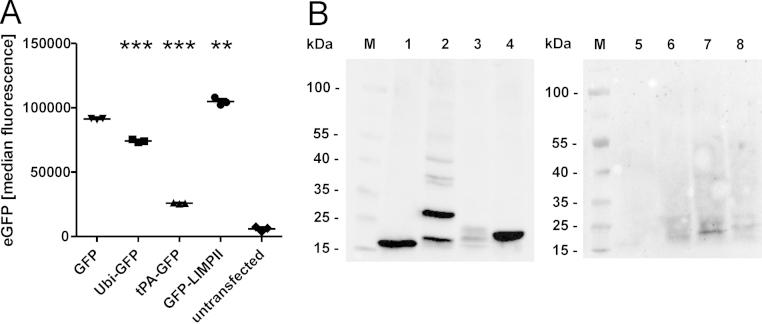


**Figure:**
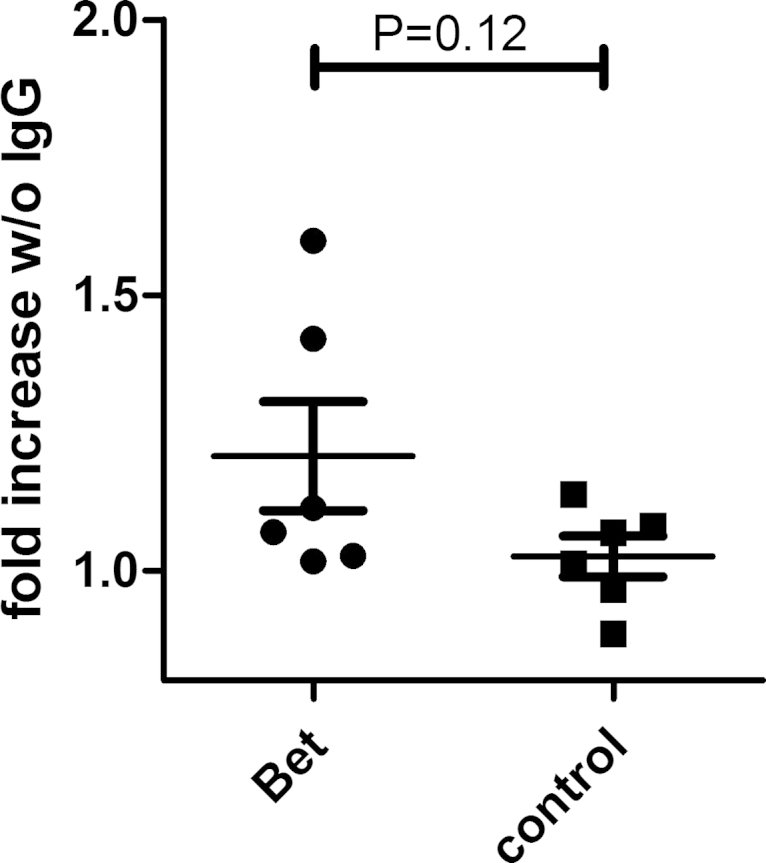


**Figure:**
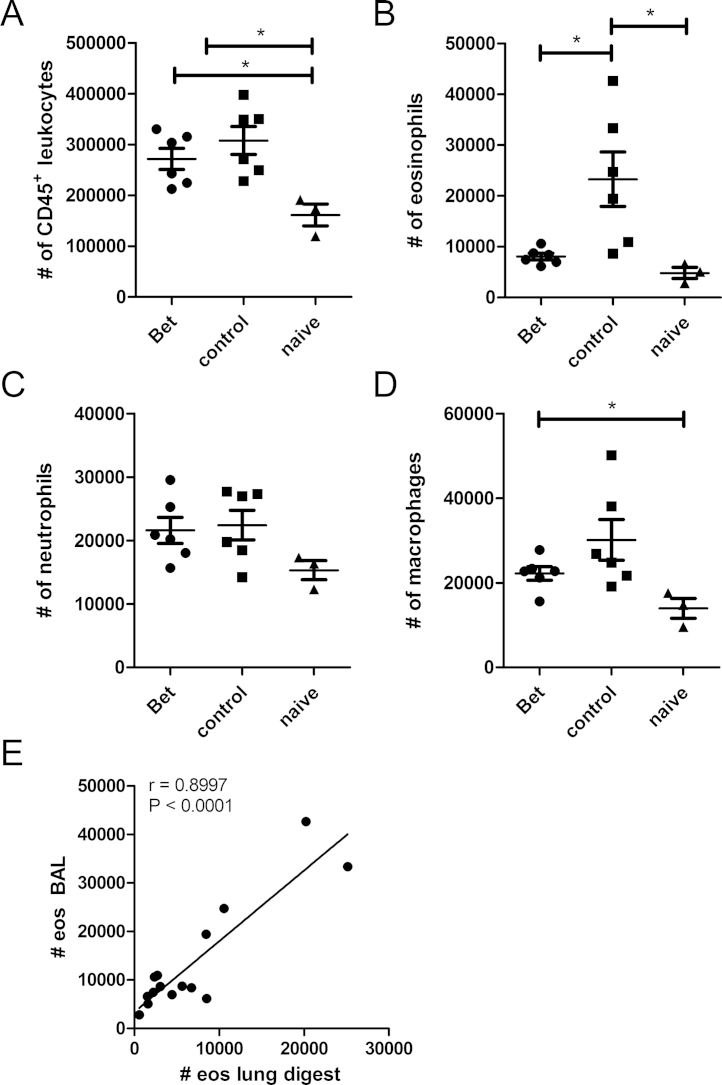


**Figure:**
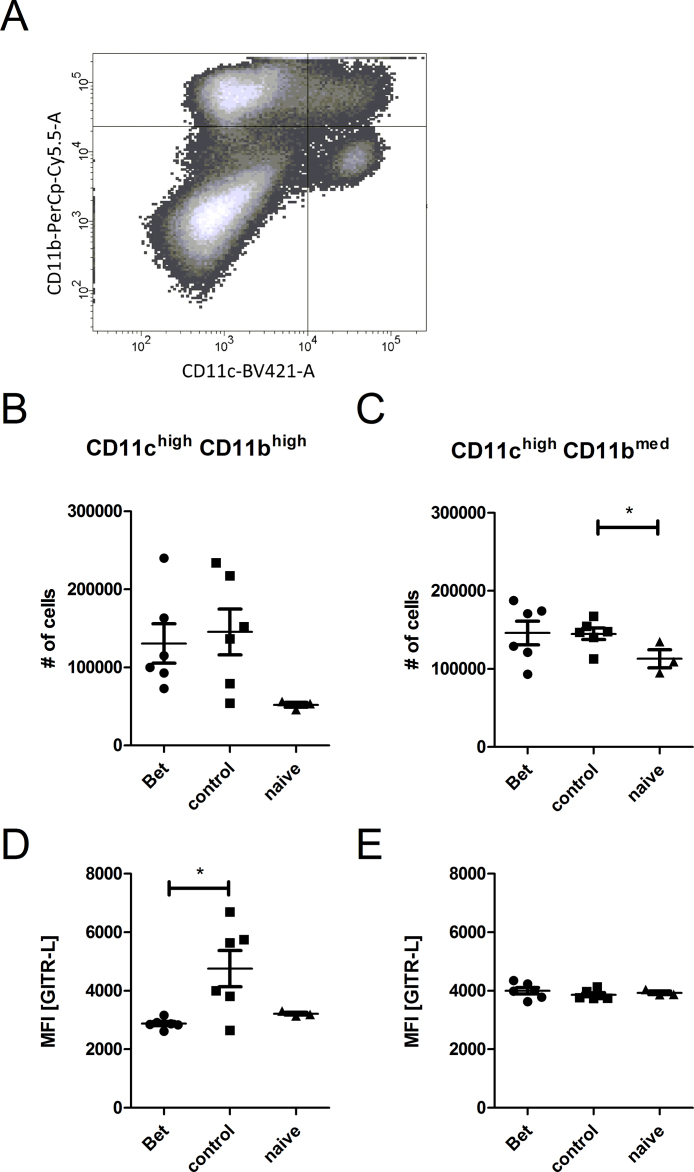


**Fig. 1:**
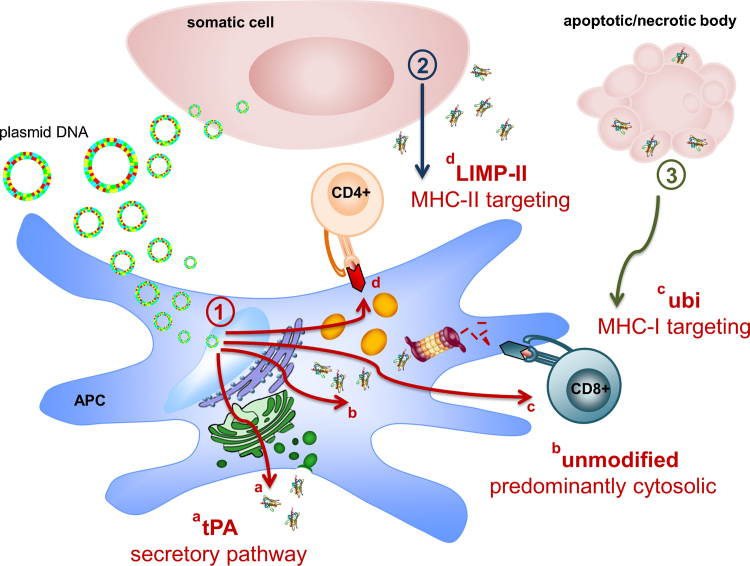
DNA vaccine-targeting strategies. After entering the nucleus (1), the plasmid DNA is transcribed and differentially processed, depending on the respective modification. (a) The 5 ‘attached tPA signal sequence leads to shuttling of the vaccine-derived Bet to the exterior of transfected cells via the Golgi apparatus, while (b) the unmodified genetic information is translated into the cytosol, leading to vaccine-derived endogenous peptide presentation on MHC-I molecules. (c) Ubiquitin attachment feeds the translated protein into the polyubiquitination pathway thereby specifically targeting peptides to MHC-I. (d) In contrast, LIMPII peptide attachment promotes the antigenic transport to lysosomes that facilitate peptide presentation on MHC-II. Along with direct transfection of both, resident immunocompetent as well as somatic cells, (2) the engulfment of secreted vaccine-derived antigens, that have been shed from transfected cells, enforce peptide processing within the endocytic pathway, or, (3) MHC-I cross-presentation of cell-associated exogenous antigens, e.g. by engulfment of transfected and apoptotic cells, are potential modes of neoantigen presentation to the immune system.

**Fig. 2:**
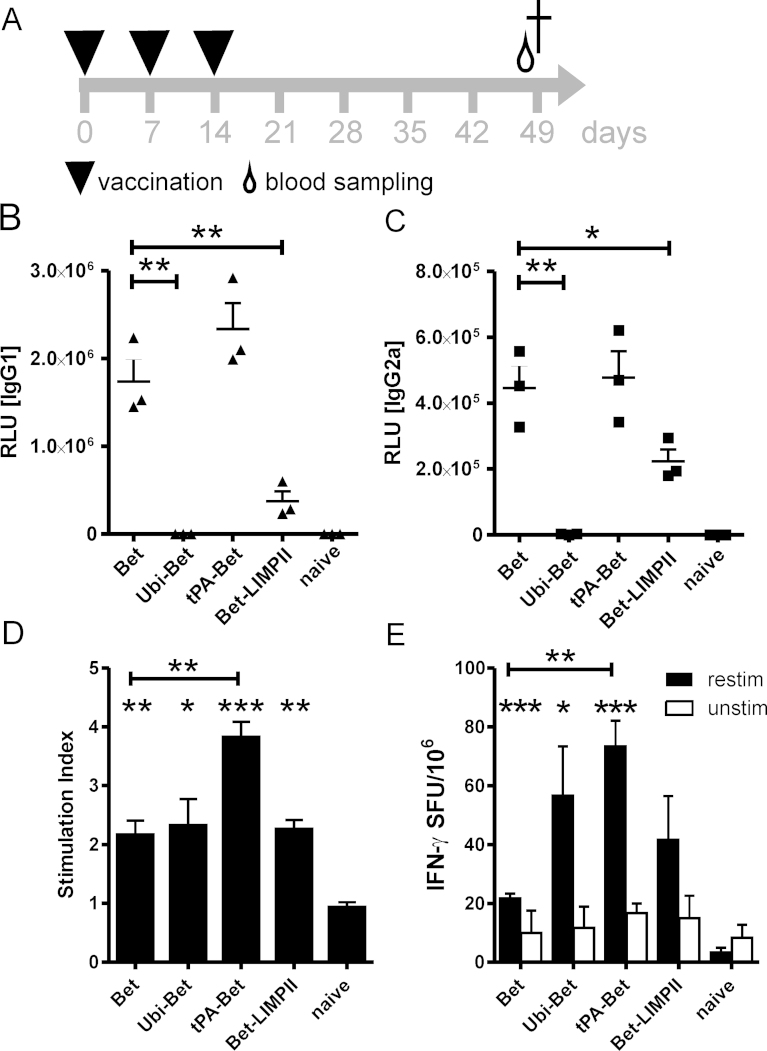
Bet-specific humoral and cellular immune responses upon i.d. genetic vaccination. (A) Schematic overview of the experimental schedule. Mice were i.d. immunized (triangle) in weekly intervals and blood samples (drop) were taken at day 49 after initial immunization. Bet-specific IgG1 (B) and IgG2a (C) antibody levels 5 weeks after the final vaccination were determined by luminescence-based ELISA. Depicted are results at a final sera dilution of 1:1000. (D) Proliferation of in vitro Bet re-stimulated splenocytes was assessed via ^3^H thymidin incorporation and the number of IFN-γ spot forming units (SFU) was determined by ELISPOT assay (E). Data are shown as means ± SEM (*n* = 3). *P < 0.05; **P < 0.01; ***P < 0.001 compared to naïve animals or as indicated.

**Fig. 3:**
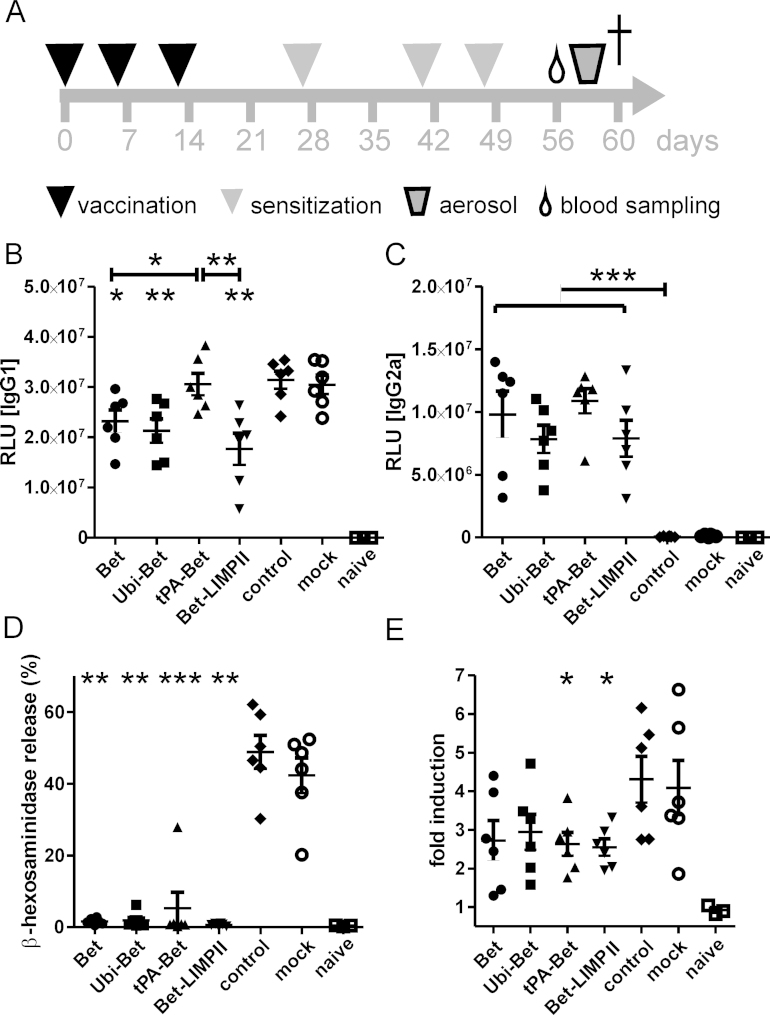
Humoral immune profile after vaccination and sensitization. (A) Schematic overview of the experimental schedule. Mice were i.d. immunized (black triangle) three times in weekly intervals and sensitized (gray triangle) for three times, before blood samples (drop) were taken at day 56 after initial immunization. After three consecutive allergen inhalation challenges (gray trapezium), lung resistance/compliance was measured and mice were sacrificed (cross). Bet-specific IgG1 (B), IgG2a (C) and IgE (D) antibody levels 1 week after sensitization (day 56) were determined via luminescence-based ELISA (IgG1, IgG2a) or RBL assay (IgE). Control animals received sham immunizations (empty pCI vector; mock) or no pre-vaccination (control) prior to Bet protein sensitization. Sera were diluted 1:1000 for ELISA (B and C) and 1:50 for RBL (D). For BAT, whole blood was ex vivo stimulated with Bet protein. Data are displayed as fold induction of up-regulated CD200R of antigen-stimulated vs. un-stimulated basophils. Data are shown as means ± SEM (*n* = 6). *P < 0.05; **P < 0.01; ***P < 0.001 compared to control group or as indicated.

**Fig. 4:**
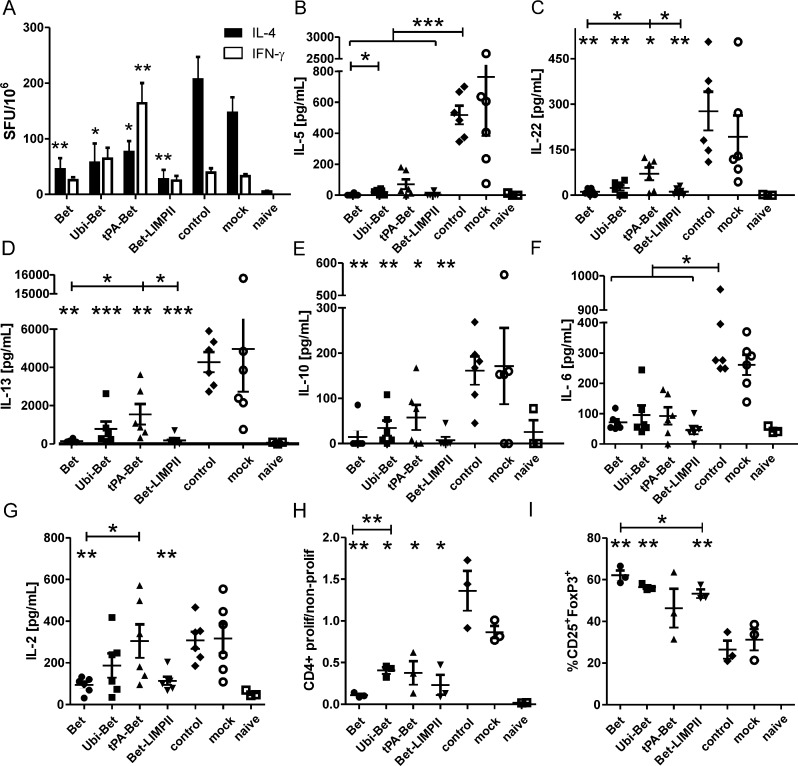
Cellular proliferation and cytokine responses of Bet re-stimulated splenocytes from pre-vaccinated animals, sham immunized (mock), or non-immunized (control) animals after sensitization. (A) Numbers of IL-4 and IFN-γ secreting splenocytes (ELISPOT) as well as an extensive panel of other cytokines (B–G) released from Bet re-stimulated splenocytes (FlowCytomix) were measured. (H) CFSE-based analysis of proliferating CD4+ T cells, given as fraction of proliferating to non-proliferating cells as well as (I) the percentage of CD25+ Foxp3+ of proliferating CD4+ T cells are displayed. Data are shown as means ± SEM (*n* = 6 or 3). *P < 0.05; **P < 0.01; ***P < 0.001 compared to control group or as indicated.

**Fig. 5:**
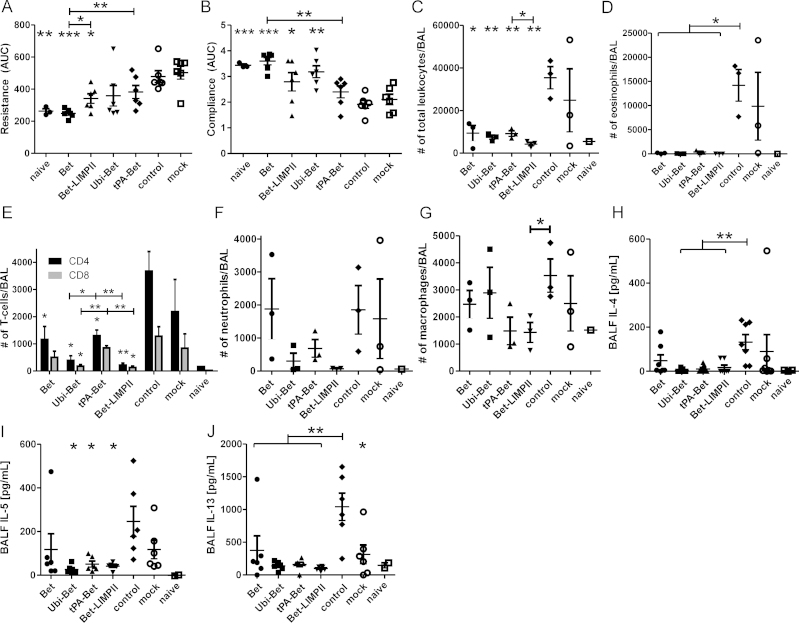
Airway hyperresponsiveness and BAL analysis of Bet pre-vaccinated, sham-immunized (mock), or non-immunized (control) animals following sensitization and airway challenge. AHR was assessed after Bet inhalation by measurement of lung resistance (A) and dynamic compliance (B). Cellular composition of BAL (C–G) was analyzed via flow cytometric analysis and BALF cytokine levels (H–J) were assessed by FlowCytomix. Both assays are presented as individual data points and/or means ± SEM (*n* = 6 or 3). AUC, area under curve; *P < 0.05; **P < 0.01; ***P < 0.001 compared to control group or as indicated.
