# Supplementary material for: The influence of antigen targeting to sub-cellular compartments on the anti-allergic potential of a DNA vaccine
Source: Vaccine. 2013 Dec 9;31(51):6113–21. doi: 10.1016/j.vaccine.2013.08.005 (PMC3898268; doi:10.1016/j.vaccine.2013.08.005)
Supplement: Supplementary file 1 [file mmc6.pdf]

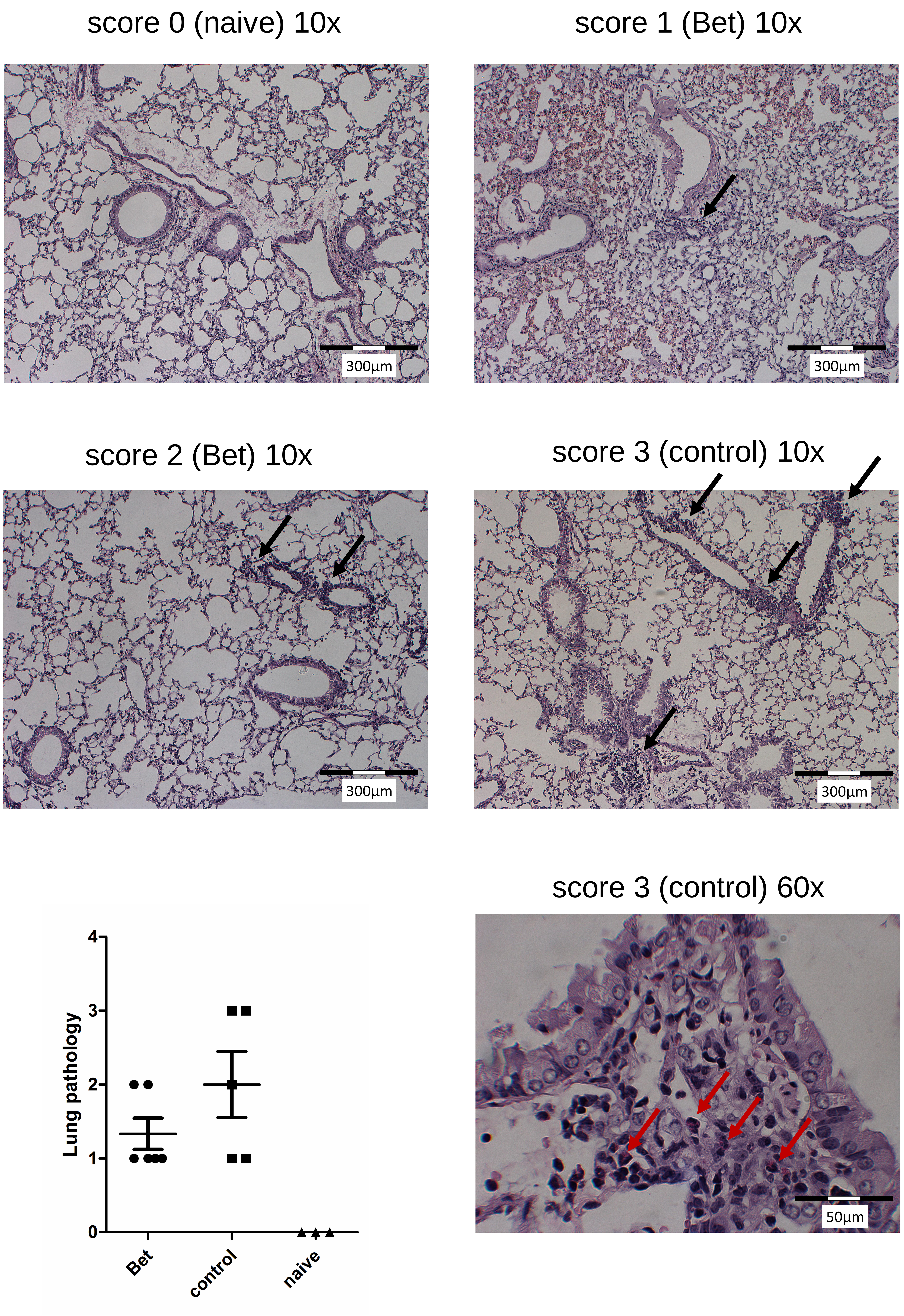
score 0 (naive) 10x
300µm
score 1 (Bet) 10x
300µm
score 2 (Bet) 10x
300µm
score 3 (control) 10x
300µm
score 3 (control) 60x
50µm
Lung pathology
0
1
2
3
4
Bet
control
naive
