# Supplementary material for: The influence of antigen targeting to sub-cellular compartments on the anti-allergic potential of a DNA vaccine
Source: Vaccine. 2013 Dec 9;31(51):6113–21. doi: 10.1016/j.vaccine.2013.08.005 (PMC3898268; doi:10.1016/j.vaccine.2013.08.005)
Supplement: Supplementary file 2 [file mmc7.docx]

# Supplementary figure captions

Suppl. Fig.1. Vector maps and AA sequences of Bet-targeting constructs. The superscripted numbers indicate the position in the full-length protein of the adjacent AA. Any additional AAs inserted as a result of the cloning process are shown in lowercase. The start and stop codons are depicted as grey boxes. The asterisk in the AA sequences represents a stop codon. LIMPII comprises 20 AAs from the C terminus of LIMPII full length protein. Ubiquitin is 76 AAs and human tPA is 22 AAs in size. CMV-IE= Cytomegalovirus immediate-early enhancer/promoter region; SV40 late poly A= SV40 late polyadenylation signal.

Suppl. Fig.2. Targeting efficacy of eGFP and Bet encoding constructs. BHK-21 cells were transfected with pCI-Bet, pCI-GFP, or the respective targeting variant. A) Protein expression 24h after transfection depicted as individual data points of GFP transfected wells. Statistical significance was calculated compared to pCI-GFP: * P<0.05, ** P<0.01, *** P<0,001. B) The lysates (1-4) and the supernatants (5-8) of Bet (1&5), Ubi-Bet (2&6), tPA-Bet (3&7), Bet-LIMPII (4&8) transfected cells were analysed after 12h by western blot analysis. M = molecular weight standard.

Suppl. Fig.3. Analysis of blocking antibodies induced upon vaccination. To assess the function of allergen-specific antibodies induced upon vaccination, sera of pre-vaccinated (Bet) and sensitization controls (control) were subjected to BAT analysis with or without the presence of each samples` respective antibody-containing plasma and further stimulated with Bet protein *ex vivo*. The presence of blocking IgG was detected by calculating the fold increase in the MFI of CD200R in the washed sample, by dividing the CD200R MFI of the washed sample by that of the unwashed sample. Data are displayed as fold increase of up-regulated CD200R of washed vs. un-washed basophils. Data are shown as means±SEM (n=6).

Suppl. Fig.4. Leukocyte populations in collagenase digested lung tissue. Total numbers of CD45+ cells (A), eosinophils (B), neutrophils (C), and alveolar macrophages (D) in single cell suspensions from the right lung lobes of pre-vaccinated mice (Bet), sensitization controls (control), or naïve mice were assessed by flow cytometry. E) Correlation between eosinophil numbers recovered from BALF and from lung digests. * P< 0.05.

Suppl. Fig.5. GITR-L expression on lung antigen presenting cells. Single cell suspensions from collagenase digested lungs of pre-vaccinated mice (Bet), sensitization controls (control), or naïve mice were prepared, and magnetic-bead sorted CD45+ cells were analysed for CD11b, CD11c, and GITR-L expression by flow cytometry. Gating strategy of CD11b^high^ CD11c^high^ cells (upper right quadrant) and CD11b^med^ CD11c^high^ cells (lower right quadrant) is shown in a density blot (top). Total numbers of the respective cell populations recovered from digests of the right lung lobes are shown in the middle panels. The respective expression of GITR-L is presented as mean fluorescence intensity (MFI) in the bottom panels. * P< 0.05.

Suppl. Fig.6. Lung pathology scores of HE-stained paraffin sections. Left lung lobes from pre-vaccinated mice (Bet), sensitization controls (control), or naïve mice were fixed and 2µm paraffin sections were prepared, HE stained, and analysed by light microscopy. Representative sections are shown at an original magnification of 10x or 60x. Perivascular and peribronchial infiltrates are indicated by black arrows. Red arrows denote eosinophilic granulocytes in the infiltrate.

# Supplementary methods

### Bet v 1.0101 and eGFP targeting variants

All constructs were cloned into the pCI mammalian expression vector (GenBank/EMBL number: [U47119](http://www.ncbi.nlm.nih.gov/nuccore/http:/www.ncbi.nlm.nih.gov/nuccore/U47119)). The cloning strategies for pCI-Bet [[1](#_ENREF_1)], which contains the coding sequence of the major birch pollen allergen Bet v 1.0101 (Bet) (Suppl.Fig.1A), as well as for pCI-Ubi-Bet [[2](#_ENREF_2)], which contains the first 76 amino acids (AA) of ubiquitin (with Gly76 changed to Ala76 to diminish the rate of cleavage) attached to the 3`end of Bet (Suppl.Fig.1B), have been previously described. pCI-LIMPII was designed using two overlapping oligos (LIMPII sense: CGCGTGGCCAGGGCAGCATGGACGAGGGGACCGCCGACGAGCGCGCCCCCCTGATCCGCACCTGAT; LIMPII antisense: CTAGATCAGGTGCGGATCAGGGGGGCGCGCTCGTCGG CGGTCCCCTCGTCCATGCTGCCCTGGCCA), (Suppl.Fig.1C), encoding the 20AA tail of the LIMPII protein (RGQGSMDEGTADERAPLIRT) [[3](#_ENREF_3)]. The adaptor was ligated into pCI-tPA/Bet/ESO, substituting ESO for LIMPII (pCI-tPA/Bet-LIMPII) and subsequently transferred into pCI vector (pCI-Bet-LIMPII). pCI-tPA-Bet was constructed by ligating pCI-encoded Bet into pCMV-MCS3 [[4](#_ENREF_4)] (Suppl.Fig.1D).

For the eGFP targeting variants, pEGFP-N1 encoded eGFP was transferred into the pCI vector (pCI-GFP), (Suppl.Fig.1A). All GFP-targeting variants were constructed analogous to the cloning strategies used for Bet variants (Suppl.Fig.1B-D). Endotoxin levels were <0.1EU/µg of DNA as determined by Limulus amebocyte lysate (LAL) assay (Pyroquant Diagnostik, Germany). Briefly, endotoxin levels contained in samples were quantified via measuring the level of turbidity using a standard curve ranging from 1.0 to 0.0625 EU/ml generated with the supplied endotoxin NP-4. Endotoxin-free LAL-reagent water served as negative control. 50µL sample were added to 50µL LAL reagent in a 96-well flat-bottom microtiter plate and turbidity, at OD 405 nm, was assessed in a kinetic fashion over a period of 30 min at 37°C in an Infinite M200 Pro plate reader (Tecan, Salzburg, Austria). Endotoxin concentrations were calculated by subtracting the mean OD of the negative control from all other readings and plotting the sample OD reads against the standard endotoxin curve.

### In vitro expression, flow cytometry and western blot analysis

To determine levels of protein expression from the respective vectors *in vitro*, GenJetTM (SigmaGen Laboratories) transfections were performed according to the manufacturer`s instructions. Briefly, 8x10^4^cells/mL BHK-21 cells (ATCC number: CCL-10) in 500µL DME-medium supplemented with 5%FCS, 2mM L-glutamine, 1mM sodium pyruvate, 1M HEPES, 100U/mL penicillin and 0.1mg/mL streptomycin were cultivated in 48-well cell culture flat-bottom plates (BD Falcon™) overnight. 1 h prior to transfection, cells received fresh medium. For transfections, 0.375μg pCI-Bet, pCI-Ubi-Bet, pCI-Bet-LIMPII, or pCI-tPA-Bet were diluted in 15μL of serum free DMEM. 0.75μL of GenJetTM was also diluted in 15μL serum free DMEM, directly added to the plasmid DNA and incubated for 15 min at room temperature (RT). 30µL were added carefully to each transfection well and 5 h later, cells were again placed in fresh complete medium. To analyse protein production, samples of supernatants or cell lysates were analysed by reducing SDS PAGE (10%) at 140V for 2 h, followed by western blot analysis. Briefly, the SDS-gel was blotted at 5V for 45 min and then at 10V for 15 minutes. After blocking of the membrane with TBS blocking buffer containing 0.1% Tween20 and 5% milk powder for 1h at 4°C, a polyclonal anti-Bet antibody (affinity purified from pooled sera of mice immunized with recombinant protein adsorbed to alum) diluted 1:1000 was added over night. After washing steps using TBS blocking buffer, a HRP-labeled goat anti-mouse IgG1 (AbD Serotec) diluted 1:1000, was added for 1h at 4°C. After multiple washing steps with blocking buffer and PBS, Immun-Star^TM^ WesternC^TM^ Chemiluminescent Kit substrate (BioRad, Munich, Germany) was added according to the manufacturer`s instructions and the blot was analyzed on a western blot imager (BioRad, Munich, Germany).

For GFP targeting variant transfections, BHK-21 cells (1x10^5^ cells/mL) were transfected with 1µg plasmid in 1mL DME-5 medium in a 24-well cell culture flat-bottom plate (BD Falcon™, NJ, USA) . 1 h prior to transfection, cells received fresh medium. For transfection, 1µg plasmid DNA was diluted in 50µL serum-free DMEM. 2µL GenJet was also diluted in 50µL serum free DMEM and added to the diluted plasmid DNA and incubated for 15 min at RT. 100µL were added carefully to each transfection well and 5 h later cells were again placed in fresh complete medium. Cells were harvested 6h and 24h after transfection to be analysed on a FACSCanto II flow cytometer.

### Lymphocyte cultures

Splenocytes were cultured as described previously [[4](#_ENREF_4)]. Briefly, mice were sacrificed and spleens were minced and sedimented for 5 min in 1 mL MEM medium. For lysis of red blood cells, single cell suspensions were added to ACK lysing buffer for 7 min followed by two washing steps in 10 mL MEM. Cells were re-suspended in MEM supplemented with 1% (v/v) heat-inactivated fetal calf serum, 100U penicillin, and streptomycin/ml, 2mM L-glutamine, 1mM sodium pyruvate, 2 µM of 2-mercaptoethanol, 20mM Hepes, and 1x non-essential amino acids. 50µL/well splenocyte solution was distributed into 96-well, flat-bottomed tissue culture plates (Becton–Dickinson, Franklin Lakes, NJ) at a density of 2x10^5^ cells/well. Recombinant antigen was added in 50µL supplemented MEM medium to a final concentration of 20 µg/mL and plates were incubated for 72 h at 37°C, 95% relative humidity, 7.5% CO_2_. Wells stimulated with medium alone served as negative controls. After 3 days, the supernatants of wells were harvested and stored at -20°C before they were subjected to cytokine analysis via the murine Th1/Th2/Th17/Th22 13-plex FlowCytomix multiplex kit (eBioscience) combined with the mouse GM-CSF FlowCytomix simplex kit (eBioscience). IL-4 and IFN-γ were additionally measured via enzyme-linked immunosorbent spot (ELISPOT) assay: Lymphocytes prepared as aforementioned were cultured in anti-IFN-γ or anti-IL-4 coated (4 µg/mL) ELISPOT plates (Merck Millipore) with or without 20µg/mL Bet v 1.0101 antigen for 24 hours as described for proliferation cultures. Cytokines were detected with biotinylated mAbs (2 µg/mL) followed by streptavidin-horseradish peroxidase (1:1000). Matched antibody pairs and streptavidin-horseradish peroxidase were purchased from eBioscience. The assay was developed by using 3-amino-9-ethyl-carbazole substrate (Acros, Geel, Belgium).

The antigen-dependent proliferation of splenocytes was studied by CFSE staining of starting cultures followed by flow cytometric analysis after 7 days of stimulation with Bet. Briefly, cells were surface stained with anti-CD4 and anti-CD25 (eBioscience), followed by fixation, permeabilization, and intracellular staining with a FoxP3 staining kit (eBioscience) according to the manufacturer’s protocol. Live CD4+ splenocytes were gated according to FSC/SSC characteristics and CD4 expression and the numbers of proliferated (CFSE^low^) and non-proliferated (CFSE^high^) cells were determined. Proliferation was expressed as the number of non-proliferated cells divided by the number of proliferated cells. Additionally, the percentage of FoxP3+CD25+ T cells of all proliferated CD4+ cells was assessed.

Alternatively, after 2 days culture, wells were pulsed with 0.5 µCi [^3^H]thymidine added in a volume of 20 µL culture medium for another 20 h. Cells were harvested with a cell harvester (Cell Harvester 96 Mach IIIM, Tomtec, CT, US). Cellular proliferation was assessed by measuring thymidine incorporation in a scintillation counter (Wallac MicroBeta TriLux, Perkin–Elmer, Finland). Stimulation indices were calculated from the averages of three replicate wells according to the equation: [antigen_cpm_/medium_cpm_].

### Luminescence-based ELISA assay

Antigen-specific IgG1 and IgG2a were determined by luminescence-based ELISA assay as previously described [[4](#_ENREF_4)]. Briefly, white 96-well high bind immunoplates (Greiner, Kremsmünster, Austria) were coated with 50µL/well recombinant antigen [1μg/mL] diluted in PBS for 24h. Next, plates were washed with PBS/0.1% Tween20 using the 96PW automatic ELISA-plate washing device (Tecan, Salzburg, Austria) and blocked with 200μL blocking buffer for 1 hour at RT. Afterwards, plates were washed again and different dilutions (ranging from 1:50 – 1:100.000) of the sera in blocking buffer were incubated for 1 hour at RT, washed again, and 50μL of a peroxidase (HRP)-conjugated detection antibody diluted 1:1000 in blocking buffer were added to the wells, recognizing either total murine IgG (BioLegend, San Diego, CA, USA) or different isotypes including IgG1 (Zymed, San Francisco, CA, USA), IgG2a (Zymed, San Francisco, CA, USA), IgG2b (Invitrogen, Carlsbad, CA, USA), IgA (Zymed, San Francisco, CA, USA) or IgE (Serotec, Düsseldorf, Germany). After incubation for 1 hour at RT, the luminometric assay was developed with Luminol diluted 1:2 in H_2_O, according to the manufacturer`s instructions (BM chemiluminescence substrate, Boehringer-Mannheim, Germany). Briefly, 50μL substrate were added to each well and chemiluminescence (photon counts/second) was determined after 3 minutes of incubation using an Infinite M200 Pro plate reader (Tecan, Salzburg, Austria).

### RBL assay

Serum IgE was measured by rat basophil leukemia (RBL) cell assay [[5](#_ENREF_5)] as previously described. RBL-2H3 cells (ATCC CRL-2256) were plated onto 96-well, flat-bottom tissue culture plates (Falcon, NJ, USA) at a density of 6x10^5^ cells/mL in 100μL RPMI 1640 supplemented with 10% (v/v) heat-inactivated fetal calf serum, 100U penicillin and streptomycin/mL, 4mM L-glutamine, 2mM sodium pyruvate, 10mM HEPES, and 100 µM of 2-mercaptoethanol and incubated over night at 37°C, 95% relative humidity, 7% CO_2_. Cells were sensitized by addition of different serum dilutions (ranging from 1:10 – 1:1200). Wells to assess background and maximum release (100%) values were left untreated. After 2h, plates were gently washed twice with 200μL Tyrode`s buffer (137mM NaCl, 2.7mM KCl, 0.5mM MgCl2, 1.8mM CaCl2, 0.4mM NaH2PO4, 5.6mM D-glucose, 12mM NaHCO3, 10mM HEPES, and 0.1% (w/v) BSA, pH 7.2) to remove unbound antibodies, followed by addition of 100µL of a 0.3 µg/mL solution of recombinant allergen in Tyrode`s buffer for 30 min to induce crosslinking of FcεR-bound IgE and subsequent degranulation of RBL cells. For determination of maximum release, 10µL of a 10% Triton X-100 solution was added to induce cellular disruption. After incubation, 50μL supernatant was removed from the wells and transferred into fresh flat-bottomed 96-well plates (Greiner, Kremsmünster, Austria), where 50μL assay solution at a final concentration of 80μM 4-Methyl umbelliferyl-N-acetyl-b-D-glucosaminide (4-MUG, Sigma, Deisenhofen, Germany) in 0.1M citrate buffer (pH 4.5) was added to each supernatant followed by incubation for 1h. The reaction was stopped via adding 100µl glycine buffer (0.2M glycine and 0.2M NaCl, pH 10.7) and fluorescence (in relative fluorescence units) was measured at λ_ex_ (360nm)/ λ_em_ (465 nm) using a fluorescence microplate reader (Spectrafluor, Tecan, Austria). Results were calculated according to the equation: [(experimental_rfu_-background_rfu_) / (maximum_rfu_ - background_rfu_)] x 100.

### Invasive lung measurement

Airway hyperreactivity (AHR) was measured after antigen-specific vaccination, sensitization and allergen inhalation via invasive measurement of pulmonary resistance and dynamic compliance using a FinePointe™ RC system for mice (Buxco, Winchester, UK), following the manufacturer's instructions. Mice were anesthetised by i.p. injection of 140µL/25g body weight Ketamine and Xylazine [20mg/mL Ketamine-HCl, 2mg/mL Xylazine-HCl] diluted in 0.9% NaCl. The trachea was cannulated and connected to the ventilator. Via an oesophageal cannula, the transpulmonary pressure was monitored and recorded. After recording baseline, mice received an aerosolized 0.9% NaCl solution containing increasing dosages of methacholine (5, 10, and 20 mg/mL), and flow and pressure signals were analysed using BioSystem XA software (Buxco, Winchester, UK). Values are shown as the integrated area under the dose response curve (AUC).

### Cellular infiltrate and cytokine profile in bronchoalveolar fluids

BALs were performed as previously published [[6](#_ENREF_6)]. Briefly, mice were sacrificed after resistance/compliance measurement and tracheae were dissected for insertion of a flexible tubing to flush the animal`s lungs twice with 1 mL ice-cold PBS. The lavages of each animal were pooled after they were flushed through a 40μm cell strainer (Becton Dickinson, New Jersey, USA). Afterwards, BALs were centrifuged at 1200 rpm for 10 minutes at 4°C and the supernatants were collected and stored at –20°C for subsequent cytokine measurement. Cells were stained with 20μL of an antibody mixture containing CD45-PE-Cy7 (clone 30-F11, BD Pharmingen), Gr1-APC (clone RB6-8C5, Biolegend), CD8-FITC (clone 53-6.7, eBioscience), CD4-APC-eFluor780 (clone RM4-5, eBioscience), and SiglecF-PE (clone E50-2440, BD PharMingen). After staining, cells were resuspended in 150µL FACS buffer (PBS, 1% BSA, 2mM EDTA) and 100µL were analysed on a FACSCanto II flow cytometer. Specific cell numbers per BAL were calculated according to the analysed volume (i.e. measured cell number x 1.5). Eosinophils were distinguished from other leukocyte populations by their SiglecF^high^CD45^med^ phenotype, while neutrophils displayed Gr1^high^CD45^med^ staining. The cytokine profile in BAL supernatants was determined using the murine Th1/Th2/Th17/Th22 13-plex FlowCytomix multiplex kit (eBioscience, San Diego) according to the manufacturer`s protocol.

### Cellular composition of lung tissue

Following bronchoalveolar lavage, in some experiments lungs were perfused by injecting 10mL cold PBS into the right ventricle until the lungs were cleared from all blood. Subsequently, lungs were inflated again, by injecting 1mL of PBS via the tracheal tubing, and carefully removed and placed in a petri dish containing PBS. Left and right lung lobes were separated and the right superior, middle and inferior lobes were digested as follows, while the left lobe was fixated for 24h in PBS, 4% formalin for histological analysis. Right lung lobes were minced in 2mL RPMI, supplemented with 0.28U/mL Liberase TM (Roche), 30µg/mL DNAse I (Roche), and 0.1% Hyaluronidase (Sigma) and were incubated on a shaker at 150rpm at 37°C for 1h. The monodisperse supernatant was transferred into a 15mL tube and put on ice. 2mL fresh digestion buffer was added to the remaining tissue and the samples were homogenized by shearing the solution using a 21 gauge needle until no visible clumps remained. The solution was then incubated on the shaker for 30min at 37°C. The suspensions were joined with the samples on ice and 80µL 0.5M EDTA (Sigma) were added. Samples were centrifuged through a 100µm cell strainer (BD Biosciences). To purify leukocytes from the digested lungs, samples were stained with anti-mouse CD45.2-biotin antibody (clone 104, Biolegend) and subsequently incubated with 30µl of streptavidin coupled BD IMag™ beads (BD Biosciences). CD45+ cells were then magnetically separated from lung tissue following the manufacturer’s protocol.

Purified CD45+ cells were then stained either with CD11b-PerCp/Cy5.5 (clone M1/70, Biolegend), CD11c-BV421 (clone N418, Biolegend), and GITRL-PE (clone YGL-386, eBioscience) or SiglecF-PE (BD Biosciences) and Gr1-APC (clone RB6-8C5, eBioscience), and >300.000 events were analysed on a FACS Canto II flow cytometer. Alveolar macrophages were identified as SiglecF+ cells [[7](#_ENREF_7)] and were clearly distinguishable from SiglecF+ eosinophils by their higher FSC and higher expression of CD45.

**Lung histology**

Lungs were ﬁxed in 4% formalin at 4°C overnight and embedded in parafﬁn. Tissue sections of 2 µm were stained with hematoxylin and eosin (HE) dye or periodic acid–Schiff (PAS) by using standard

protocols. All sections were analysed by means of light microscopy by an investigator who was blinded to the treatments. Severity of inﬁltrates in HE sections was scored as follows: score 0 – no infiltrates; score 1 – very rare infiltrates around blood vessels; score 2 – some infiltrates around blood vessels; score 3 – infiltrates around blood vessels and beginning infiltrates around bronchioles.

### Basophil activation test (BAT)

The basophil activation of murine whole blood samples was performed as described before [[8](#_ENREF_8)]. In short, whole blood (50µL) was diluted with 50µl RPMI 1640 (PAA, Pasching, Austria) containing 200µg/mL heparin, and incubated with 40µg/mL Bet v 1.0101, with 2µg/mL anti-IgE (clone 23G3, eBioscience) as positive control, or left untreated as negative control for 2 h at 37°C and 7% CO_2_. Cells were washed and centrifuged at 500g for 5 min prior to surface staining using anti-CD200R3 (clone Ba103, Hycult biotech), anti-CD49b (clone DX5, BioLegend), anti-CD200R (clone OX110, eBioscience), and anti-CD45 (clone 30-F11, BD Biosciences). After incubation, cells were washed twice with PBS/1%BSA/2mM EDTA and analysed by flow cytometry. Data are shown as fold induction by dividing the median fluorescence intensity (MFI) of CD200R in the Bet v 1 re-stimulated sample, by the MFI of CD200R in the negative control sample, respectively.

In some experiments, blood samples were washed 3 times with RPMI to remove any potential blocking IgG in the plasma prior to incubating the cells with 20µg/mL Bet v 1.0101. The presence of blocking IgG was detected by calculating the fold increase in the MFI of CD200R in the washed sample, by dividing the CD200R MFI of the washed sample by that of the unwashed sample.

# Supplementary references

[1] A. Hartl, J. Kiesslich, R. Weiss, A. Bernhaupt, S. Mostbock, S. Scheiblhofer, C. Ebner, F. Ferreira, J. Thalhamer, Immune responses after immunization with plasmid DNA encoding Bet v 1, the major allergen of birch pollen, The Journal of allergy and clinical immunology, 103 (1999) 107-113.

[2] R. Bauer, S. Scheiblhofer, K. Kern, C. Gruber, T. Stepanoska, T. Thalhamer, C. Hauser-Kronberger, B. Alinger, T. Zoegg, M. Gabler, F. Ferreira, A. Hartl, J. Thalhamer, R. Weiss, Generation of hypoallergenic DNA vaccines by forced ubiquitination: preventive and therapeutic effects in a mouse model of allergy, The Journal of allergy and clinical immunology, 118 (2006) 269-276.

[3] F. Rodriguez, S. Harkins, J.M. Redwine, J.M. de Pereda, J.L. Whitton, CD4(+) T cells induced by a DNA vaccine: immunological consequences of epitope-specific lysosomal targeting, Journal of virology, 75 (2001) 10421-10430.

[4] A. Hartl, R. Weiss, R. Hochreiter, S. Scheiblhofer, J. Thalhamer, DNA vaccines for allergy treatment, Methods, 32 (2004) 328-339.

[5] R. Hochreiter, T. Stepanoska, F. Ferreira, R. Valenta, S. Vrtala, J. Thalhamer, A. Hartl, Prevention of allergen-specific IgE production and suppression of an established Th2-type response by immunization with DNA encoding hypoallergenic allergen derivatives of Bet v 1, the major birch-pollen allergen, European journal of immunology, 33 (2003) 1667-1676.

[6] M. Gabler, S. Scheiblhofer, K. Kern, W.W. Leitner, A. Stoecklinger, C. Hauser-Kronberger, B. Alinger, B. Lechner, M. Prinz, S. Vrtala, R. Valenta, J. Thalhamer, R. Weiss, Immunization with a low-dose replicon DNA vaccine encoding Phl p 5 effectively prevents allergic sensitization, J Allergy Clin Immunol, 118 (2006) 734-741.

[7] A.C. Kirby, M.C. Coles, P.M. Kaye, Alveolar macrophages transport pathogens to lung draining lymph nodes, J Immunol, 183 (2009) 1983-1989.

[8] E.E. Weinberger, M. Himly, J. Myschik, M. Hauser, F. Altmann, A. Isakovic, S. Scheiblhofer, J. Thalhamer, R. Weiss, Generation of hypoallergenic neoglycoconjugates for dendritic cell targeted vaccination: A novel tool for specific immunotherapy, Journal of controlled release : official journal of the Controlled Release Society, 165 (2012) 101-109.
